# A Retrospective Taiwanese-Population-Based Clinical Study on Determining the Efficacy and Safety of Disposable Circumcision Anastomat

**DOI:** 10.3390/jcm11206206

**Published:** 2022-10-21

**Authors:** An-Chi Chou, Chun-Yo Laih, Fang-Yu Ku

**Affiliations:** 1 Department of Urology, Taipei Medical University Hospital, Taipei 11031, Taiwan; 2 Department of Urology, China Medical University Hospital, Taichung 404327, Taiwan

**Keywords:** circumcision, disposable circumcision suture device, diabetes mellitus, HbA1C

## Abstract

Traditional approaches for male circumcision are tedious and could lead to complications such as peri-/postoperative pain, bleeding, and infection. Thus, for the first time, we investigated the surgical outcomes of recently the discovered Disposable Circumcision Anastomat Type A (Dongguan ZSR Biomedical Technology Company Ltd., China), a disposable circumcision suture device (DCSD), in terms of the operation time, surgical complexity, safety, satisfaction, and aesthetic outcomes and most importantly the prognostic factors for postoperative infection. A total of 394 individuals were circumcised, with a mean age, body mass index (BMI), stretched penile length (SPL), and penile circumference of 30.1 ± 7.05 years, 25.47 ± 4.73, 10.12 ± 1.61, and 7 ± 0.73 cm, respectively. Associated comorbidities included diabetes mellitus (6.09%), hypertension (2.03%), gout (1.02%), end-stage renal disease (ESRD, 0.25%), and HIV (0.25%). The mean operation time, average postoperative bleeding, and wound infection rate was 31.4 ± 9.96 min, 2.54%, and 9.39%, respectively. The mean VAS postoperative pain scores at D0 and D1 were 4.4 ± 2.4 and 1.9 ± 1.6, respectively. Moreover, 1.27% of subjects required reoperation, and a 2.03% rate of instrument malfunction was noted. The significant factors associated with the post-operative infection group were age (*p* = 0.0313), BW (*p* = 0.0081), BMI (*p* = 0.0026), penile circumference (*p* = 0.0343), and DM (*p* ≤ 0.001). Multivariate analyses revealed only DM as a statistically significant factor (*p* < 0.001). Our box–whisker plot revealed no significant difference between the HbA1c level of infection (Hb1Ac = 7.77 ± 1.39) and non-infection groups (Hb1Ac = 6.92 ± 1.84). However, a trend of higher glycemic index in the infection group was observed. Conclusively, DSCD could be an effective and safe alternative to performing circumcision. However, in the population with advanced aging, phimosis, elevated BMI, and DM (HbA1C > 9%), users should be highly cautious due to the increased risk of infection, dehiscence, and hematoma.

## 1. Introduction

Male circumcision (MC), the surgical removal of the penile prepuce, is associated with traditional, religious, ethnic, and cultural practices [1,2,3]. It is also executed to address medical conditions such as phimosis and redundant prepuce. Some additional advantages, such as the control of sexually transmitted diseases (STDs), urinary tract infection, HIV/HPV infection, and lower incidences of penile and cervical cancer, are associated with circumcision [4,5,6,7].

The prevalence of MC varies according to the geographical region; however, it has been estimated that around 38.7% of the population is circumcised, the majority of which belongs to Muslim and Jewish communities [8]. Gomco clamp, Plastibell, Mogen clamp, and dorsal slit methods are widely used in neonatal circumcision [1]. A clamp is used to restrict blood flow in the penile foreskin to suppress the risk of bleeding. Circumcision is a straightforward procedure among children, infants, and young adults with rapid healing and a very low risk of bleeding [1]. Moreover, pediatric circumcision benefits supersede the associated risk if operated by a professional with proper pain management under sterile conditions [9,10,11]. MC in adults is mainly performed through sleeve resection, forceps-guided, and dorsal slit methods [12]. However, these traditional approaches are tedious and could lead to complications such as peri-/postoperative pain, bleeding, and infection [13,14,15]. Thus, novel methods and devices have been developed to overcome these adverse events to improve circumcision effectiveness with enhanced safety. Currently, several novel kits, such as the Shang ring, have been developed to lower operative time [16]; however, it leaves significant remains of the mucosal layer of the foreskin, which suppresses its efficacy in HIV prevention [17].

**Figure 1:**
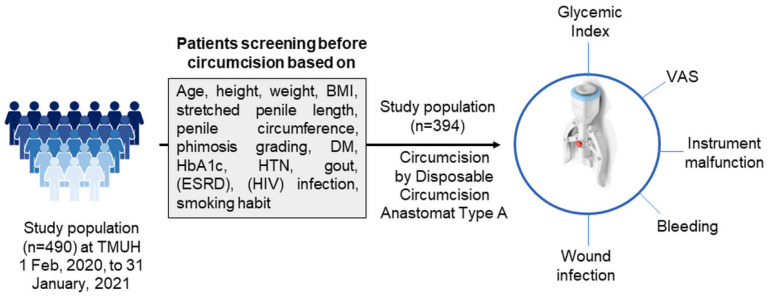
Schematics of the study design. Patients were screened based on their age, height, weight, BMI, stretched penile length, penile circumference, phimosis grading, DM, HbA1c, HTN, gout, (ESRD), (HIV) infection, and smoking habit. The outcomes of the study were measured in terms of glycemic index, wound infection, VAS, bleeding, and instrument malfunction. BMI: body mass index, DM: diabetes mellitus, HbA1C: glycated hemoglobin, HTN: hypertension, ESRD: end-stage renal disease, HIV: human immunodeficiency virus, VAS: visual analog scale.

The recently discovered disposable circumcision suture device (DCSD) is an improved intervention compared to conventional techniques, in terms of lowering surgical time, and perioperative bleeding; however, the surgical result could be further influenced by several factors, including the patient’s comorbidities causing balanitis [18,19], and an enhanced risk of infection [20]. Considering these possible complications, for the first time, this study aimed to assess the surgical outcomes of MC using Disposable Circumcision Anastomat Type A (Dongguan ZSR Biomedical Technology Company Ltd., Dongguan, China) in terms of the operation time, surgical complexity, safety, satisfaction, and aesthetic outcomes, and most importantly the prognostic factors for postoperative infection (Figure 1).

## 2. Materials and Methods

### 2.1. Patients Recruitment

A total of 490 patients with redundant prepuce or recurrent balanoposthitis received circumcision by the DCSD in our hospital from 1 February 2020 to 31 January 2021. However, we retrospectively enrolled 394 patients based on the exclusion criteria such as loss of postoperative follow-up, any missing laboratory data, or medical records.

### 2.2. Patient Demographics and Clinical Data

Patients’ age, height, weight, body mass index (BMI), stretched penile length, penile circumference, phimosis grading, diabetes mellitus, glycated hemoglobin (HbA1c), hypertension (HTN), gout, end-stage renal disease (ESRD), human immunodeficiency virus (HIV) infection, and smoking habit were recorded before the circumcision. We graded the extent of phimosis according to the scale proposed by Kikiros et al. [21]. We would also survey the patient’s HbA1c if they mentioned underlying diabetes mellitus (DM) or any clinical signs of DM. Perioperative parameters were as follows: 12 h post-op visual analog scale (VAS, marked as day 0), post-op day 1 VAS, infection, instrument malfunction, and operation time. Adverse events, including post-operative bleeding, edema, wound infection, and wound infection requiring reoperation, were recorded. Wound infection was defined as any clinical sign of wound redness, swelling, local heat, or persistent pain with or without wound dehiscence that led to a prescription of curative empiric antibiotics. Post-operative bleeding was defined as bleeding that could not stop by self-compression, and the patient returned to our emergency department or office for management. We collected all the parameters from outpatient records and surgical records.

### 2.3. Surgical Instruments and Procedures

For the circumcision device, we used the Disposable Circumcision Anastomat Type A (Dongguan ZSR Biomedical Technology Company Ltd., Dongguan, China). The DCSD exists in nine models according to different penile girths: 10, 12, 14, 16, 18, 22, 26, 30A, and 34. The device consists of a bell-shaped glans pedestal, suture staples, ring-shaped blade, handle, and shell. The appropriate size of the device was selected according to the penile circumference, using the measuring scale provided by the manufacturer. The patients were placed supine, disinfected, and received local dorsal penile nerve block injection with 2% lidocaine up to 10 mL. After anesthesia, we applied three Mosquito forceps, one under the frenulum and the others bilaterally, to extend the prepuce. The forceps were applied under the frenulum at least 1 cm away from the sulcus to ensure sufficient skin margin from the wound to the glans. The glans pedestal was inserted into the prepuce and kept in proper tension by forceps. We then constrained the foreskin with 4-0 Silk achieving a drawstring-pocket-like model. Before we tied up the suture, and the shaft was tilted to an angle of 30–45 degrees from the horizontal level to preserve the frenulum. We inserted the screw of the properly fixed pedestal into the center of the Anastomat. The knob was tightened, and the handles were clenched after removing the safety bar. We kept the handles clenched for 30 s to ensure better hemostasis. The foreskin was cut instantly with simultaneous anastomosis by the suture staples. After the procedure, we compressed the wound for five minutes, and the rubber ring was cut every 3–5 nails to prevent over-compression. If active bleeding was noted, a state suture with a 4-0 Monocryl state suture was applied. Then, the circular wound dressing was completed with a self-adhesive flexible bandage.

After the surgery, the patient received one dose of parecoxib via intramuscular injection, three days of prophylactic oral cefazolin, and 28 pills of diclofenac as self-administered pain control medication. The patients returned to our clinic in one week and one month for postoperative evaluation. The patients were instructed to return to our office or emergency department if there were any signs of wound infection or bleeding, such as progressive tenderness, swelling, or dehiscence. Once the diagnosis of wound infection was made, oral augmentin (1 gm) was prescribed for one week with an instruction to keep changing the wound dressing daily. In case of no improvement in wound dehiscence, debridement and primary closure of the wound were suggested.

### 2.4. Statistical Analysis

Data were analyzed by using SPSS 26.0 (IBM, Armonk, NY, USA). Student’s *t*-test was employed for numerical data comparison, whereas a chi-square test was conducted for categorical variables. A *p*-value less of than 0.05 was considered statistically significant.

## 3. Results

### 3.1. Demographics and Characteristics of Patients

A total of 394 individuals received DCSD-based circumcision, and their demographic characteristics are summarized in Table 1. The mean age and mean BMI of the persons were 30.1 ± 7.05 years and 25.47 ± 4.73. The mean stretched penile length (SPL) and penile circumference were 10.12 ± 1.61 and 7 ± 0.73 cm, respectively. The comorbidities associated with these participants include diseases/disorders such as diabetes mellitus (DM) (6.09%), HTN (2.03%), gout (1.02%), ESRD (0.25%), and HIV (0.25%). Notably, twenty-four individuals (6.09%) were smokers. Phimosis is a condition when the penile foreskin becomes unretractable and can be classified based on Kikiro’s grading [21]. In our study, 338 (85.79%), 14 (3.55%), and 42 (10.66%) individuals were in phimosis grades of 0–1, 2–3, and 4–5, respectively.

### 3.2. Operation Time, Pain, Postoperative Bleeding, and the Adverse Events

The mean operation time was 31.4 ± 9.96 min and the average postoperative bleeding, and the wound infection rate was 2.54% and 9.39% of the total population, respectively. The mean VAS postoperative pain scores at D0 and D1 were 4.4 ± 2.4 and 1.9 ± 1.6, respectively. We also noted that 1.27% of subjects required reoperation, while 2.03% of the individuals faced instrument malfunction during the operation. All mentioned perioperative parameters are summarized in Table 2.

**Figure 2:**
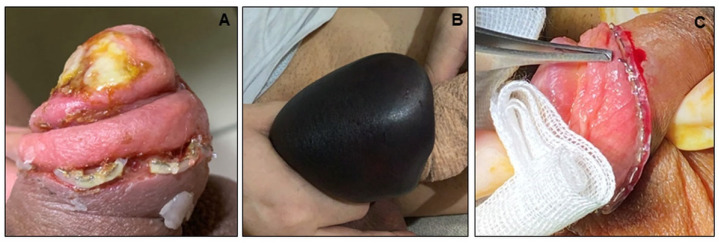
Representative photomicrographs of (**A**) infection, (**B**) hematoma, and (**C**) instrument malfunction.

Besides infection (Figure 2), we also observed a case of hematoma (Figure 2B) within the 10 bleeding cases. A few cases of instrument malfunction were also evidenced (Figure 2C).

### 3.3. Association between Wound Infection and Clinical Parameters

In total, 37 out of 394 patients were observed with wound infection. Hence, we assessed the correlation of comorbidities and perioperative parameters with the infection which is presented in Table 3. The significant factors associated with the post-operative infection group were age (32.5 ± 6.14 years vs. 29.9 ± 7.10 years, *p* = 0.0313), BW (83.94 ± 19.12 kg vs. 74.91 ± 14.82 kg, *p* = 0.0081), BMI (28.31 ± 5.78 kg/m^2^ vs. 25.17 ± 4.52 kg/m^2^, *p* = 0.0026), penile circumference (7.24 ± 0.72 cm vs. 6.97 ± 0.73 cm, *p* = 0.0343), and DM (*p* ≤ 0.001); however, other factors such as HTN, SPL, VAS, and OT remained insignificant.

### 3.4. Identifying the Risk Factor for Wound Infection

Univariate and multivariable analyses were performed to identify the risk factor for wound infection (Table 4). Univariate analyses suggest that age (*p* = 0.0327), BMI (*p* = 0.002), phimosis grading 4–5 (*p* = 0.0238), and DM (*p* < 0.001) were the significant risk factors for postoperative wound infection. However, the multivariate analyses revealed only DM as a statistically significant factor for wound infection (*p* < 0.001).

### 3.5. Impact of HbA1c on Wound Infection

**Figure 3:**
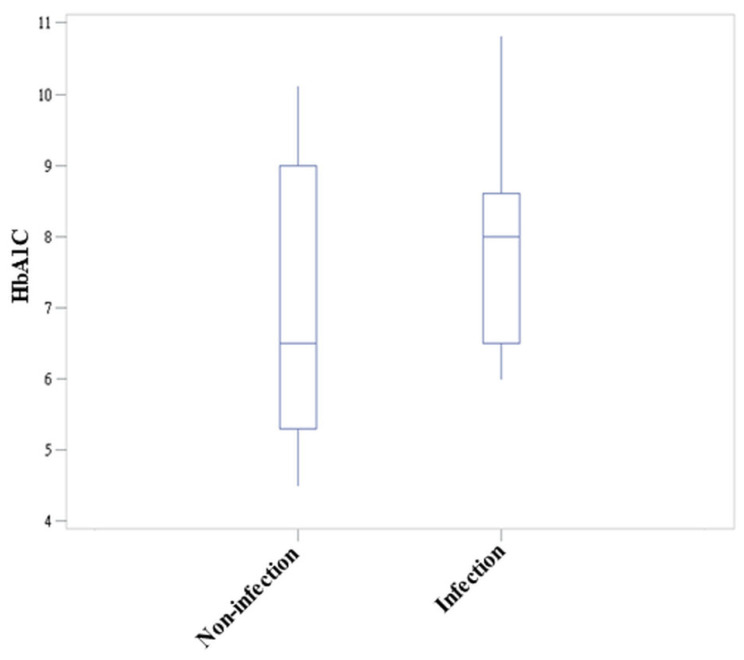
Box–whisker plot revealing the HbA1c level between infection and non-infection groups.

Hemoglobin A1C (HbA1c) represents a standard biochemical marker indicating the average glycemic control over the last 3 months, and a predictor of vascular damage [22]. DM has been demonstrated to adversely impact perioperative outcomes, with elevated rates of infection, causing further complications. Therefore, we determined the association between HbA1C levels and DCSD-based postoperative wound infection. Our box–whisker plot (Figure 3) revealed no significant difference between the HbA1c level of infection (Hb1Ac = 7.77 ± 1.39) and non-infection groups (Hb1Ac = 6.92 ± 1.84). However, a trend of higher glycemic index in the infection group was observed.

## 4. Discussion

Circumcision is one of the most common surgeries conducted all over the world, primarily due to religious, cultural, or medical reasons [23]; however, it requires a certain amount of practice to elaborate the skills. In recent years, DCSD has been developed as a minimally invasive alternative, which is safer, more effective, and easier to comprehend, while achieving satisfactory aesthetic outcomes [23,24]. No additional device is needed, and the procedure could be performed for outpatients [24,25]. Since the DCSD first came to market in 2014, it prevailed in Asia, especially in China, being the major manufacturer. After the availability of this device in Taiwan in 2019, we conducted nearly 500 cases and summarized our surgical experience with this novel technique. We intended to share our experience with the Taiwanese population in determining the prognostic factors for poor surgical outcomes, especially wound infection.

Many systemic reviews had already concluded that circumcision with DCSD is non-inferior and causes lesser complications than the conventional method. In a systematic review and meta-analysis, Huo, Z.C. et al. identified 203 related studies by 15 May 2015, and reviewed 9 out of them [2]. It was verified that the DCSD had advantages over the conventional method for the shorter operation time, faster wound healing, less intraoperative blood loss, less prepuce swelling, and lower degrees of intraoperative/postoperative pain. No differences in the incidence of wound infection, hematoma, or incision dehiscence were observed between the two groups. Another comparative study by Huang, C. et al. [26] selected 18 high-quality studies before December 30, 2016, containing 6179 patients, and concluded the same, which could not only be applied to the adults but also to the pediatric group. Further, a seminal study compared the surgical outcomes of 284 patients, aged between 7 and 16 years old [27], and confirmed the DCSD group as the most efficacious and safest choice, with a relatively lower complication rate and better cosmetic results. In an important study, the Shang Ring, a novel disposable circumcision device that consists of two concentric plastic rings, has been demonstrated to be a safe alternative with short procedure times, and less surgical skill required. However, six mild adverse events were noted, such as penile skin injuries, edema, and infection [16]. Its potential disadvantages include the need for multiple sizes and to wear it for 1 week. It also leaves significant remnants of the mucosal layer of the foreskin, which inhibits its efficacy in HIV prevention [17].

Wound infection is a major concern among surgeons. Due to the dual blood supply of the penis, wound infections are infrequent, mild, and usually resolve with topical antibiotic ointment [28]. Most of the condition manifests as local inflammatory changes, and only a few require prolonged use of oral antibiotics, and sometimes reoperation, which occurred in five of our patients. Although the infection rate was quite low across the studies, we found that certain populations were prone to this medical problem. All patients were screened for comorbidities to assess the possible prognostic factors in our study, including diabetes mellitus (DM), hypertension (HTN), and smoking. Using logistic regression to find the possible risk factors for infection, we looked further into the diabetic population.

DM is a metabolic disease prevalent in 10% of the population above 20 years of age in Taiwan. It interferes with innate and adaptive immunity [29]. In addition to this, the endothelial cells exposed to elevated blood glucose for a prolonged time become dysfunctional, leading to integrity loss and increased susceptibility to apoptosis, detachment, and circulation into the bloodstream. DM not only affects normal angiogenesis, but also disrupts proper wound healing, tissue regeneration, and the restoration of a healthy vascular system, which could be attributed to post-operative infection [30]. Martin Emily T. et al. found DM an independent risk factor for surgical site infection across different types of surgeries [31]. Even though the hyperglycemia was under control, a history of DM remained a significant risk factor in these studies. The reasons for this finding are unclear. One hypothesis is that the diabetes state contributes to the infection beyond causing hyperglycemia but also vascular changes and white blood cell dysfunction. This might explain why the long-term hyperglycemia condition (HbA1c) failed to show significance as a potential risk factor for infection in our study. In the box–whisker plot, we found a tendency that the patient was prone to infection with higher HbA1c levels. A larger patient population and more comprehensive data collection are required to better understand this finding.

The median operation time of 31 min in our study was longer than the average of other studies between 6.8–10.2 min [15,27,32,33,34,35,36,37,38]. The longer median operation time in our research may be attributed to our assigned operation time record which included time after the patient entry into the operation room and their leaving, and also the post-operative education time. However, the average surgical time was approximately 5 min. We also observed increased wound infection which could be related to age, BMI, penile circumference, phimosis, and DM. With advancing age, the innate immune response is impaired. The primary contributor to infections and delayed wound healing in the aging population could be attributed to impaired neutrophil migration, extracellular trap formation, and bactericidal mechanisms [39]. Moreover, the alterations in macrophage phagocytosis, anti-bacterial effector function, and cytokine production contribute to elevated infection risk [40]. Higher BMI due to obesity and other aberrant metabolic conditions may substantially impact the immune system, by its ability to produce inflammatory mediators such as cytokines, adipokines, and chemokines [41,42]. Penile size is a key parameter of sexual development in males, and circumference/girth is considered more important than length [43]. It has also been argued that penile size may also affect correct and consistent condom use and HIV/STI transmission [44]. However, the true correlation between the penile circumference and the poor surgical result was undetermined in our study. It may likely be attributed to mechanical malfunction resulting from a limited spectrum of device size. Sexual function was not included as a variable in our study. It has been documented that male circumcision eliminates 33–50% of the penile skin, and approximately all the penile fine-touch neuroreceptors [45]. The impact of circumcision on sexual sensation in the penis remains debated. Although it was not officially documented, there was no negative feedback reported from any of our patients [46]. Notably, the period of sexual abstinence was 1 month or until the wound healed.

A few limitations of our study were identified. First, we lacked data from the conventional method because most of the patients recruited in this study were operated on by DCSD. A comparison between the two methods was not feasible. Secondly, although we tried to collect the data as detailed as possible, some of them were still missing, considering the retrospective nature of the study. Third of all, whether the outcomes of our study could be applied to the pediatric group should be interpreted with caution because the majority of our patients were adults. Fourth, although we attempted to find the cut-off HbA1c value to determine which patient should defer their surgery to decrease the risk of infection, there was no strong evidence supporting that HbA1c < 9% was the optimal status. However, our future goal includes understanding detailed underlying mechanisms by working in collaboration with the endocrinologist. Our box–whisker plot (Figure 3) revealed no significant difference between the HbA1c level of infection (Hb1Ac = 7.77 ± 1.39) and non-infection groups (Hb1Ac = 6.92 ± 1.84). This could be attributed to the numerical values of HbA1C based on a small size of only 30 cases, which though did not reveal a significant difference, a trend of higher glycemic index in the infection group could be observed. Therefore, our future study will incorporate a greater number of patients to observe a significant difference. Further, since the patient was under local anesthesia, the pain score was not measured. We attempted to determine the pain during the first 7 days; however, after 2 days, no pain complaint was observed. Hence, no further pain assessment was carried out.

## 5. Conclusions

Our data indicate that DSCD could be an effective and safe alternative to performing circumcision. However, in the population with advanced aging, phimosis, elevated BMI, and DM (HbA1C > 9) users should be highly cautious due to the enhanced risk of infection, dehiscence, and hematoma.

## Figures and Tables

**Table 1:** Demographic data.

	*n* = 394
Age, years (Mean ± SD)	30.1 ± 7.05
Height, cm (Mean ± SD)	172.26 ± 6.75
Weight, Kg (Mean ± SD)	75.76 ± 15.47
BMI, Kg/m^2^ (Mean ± SD)	25.47 ± 4.73
SPL, cm (Mean ± SD)	10.12 ± 1.61
HbA1C * (Mean ± SD)	7.23 ± 1.71
Penile circumference (Mean ± SD)	7.00 ± 0.73
DM [*n*, (%)]	24, (6.09%)
HTN [*n*, (%)]	8, (2.03%)
Gout [*n*, (%)]	4, (1.02%)
ESRD [*n*, (%)]	1, (0.25%)
HIV [*n*, (%)]	1, (0.25%)
Phimosis grading	
Grade 0–1 [*n*, (%)]	338, (85.79%)
Grade 2–3 [*n*, (%)]	14, (3.55%)
Grade 4–5 [*n*, (%)]	42, (10.66%)
Smoking [*n*, (%)]	24, (6.09%)

BMI: body mass Index, SPL: stretched penile length, DM: diabetes mellitus, HTN: hypertension, ESRD: end-stage renal disease, HIV: human immunodeficiency virus. * indicates the HbA1C data of 30 patients.

**Table 2:** Post-operative data in the general population.

	*n* = 394
# Bleeding [*n*, (%)]	10, (2.54%)
Infection [*n*, (%)]	37, (9.39%)
Instrument malfunction [*n*, (%)]	8, (2.03%)
Reoperation [*n*, (%)]	5, (1.27%)
POD0, VAS (Mean ± SD)	4.4 ± 2.4
POD1, VAS (Mean ± SD)	1.9 ± 1.6
OP time, minute (Mean ± SD)	31.4 ± 9.96

# One case of severe hematoma was observed. POD: post-operative day, VAS: visual analog scale, OP: operation.

**Table 3:** Comparative wound infection and non-infection group.

	Infection		*p*-Value *
	No (*n* = 357)	Yes (*n* = 37)	
Hypertension	6, (1.68%)	2, (5.40%)	0.1677
DM	12, (3.36%)	12, (32.43%)	<0.001
Gout	4, (1.12%)	0, (0.00%)	1.00
Phimosis grading			0.0534
Grade 0–1	311, (87.11%)	27, (72.97%)	-
Grade 2–3	12, (3.36%)	2, (5.40%)	-
Grade 4–5	34, (9.52)	8, (21.62%)	-
Reoperation	0, (0.00%)	5, (13.51%)	<0.001
Age (Mean ± SD)	29.9 ± 7.10	32.5 ± 6.14	0.0313
Height (Mean ± SD)	172.3 ± 6.91	171.9 ± 4.96	0.6434
Weight (Mean ± SD)	74.91 ± 14.82	83.94 ± 19.12	0.0081
BMI (Mean ± SD)	25.17 ± 4.52	28.31 ± 5.78	0.0026
Penile stretch length (Mean ± SD)	10.11 ± 1.63	10.20 ± 1.46	0.7601
Penile circumference (Mean ± SD)	6.97 ± 0.73	7.24 ± 0.72	0.0343
POD0VAS (Mean ± SD)	4.41 ± 2.44	4.30 ± 2.44	0.7959
POD1VAS (Mean ± SD)	1.87 ± 1.61	1.97 ± 1.80	0.7099
OP time (Mean ± SD)	31.21 ± 9.85	33.35 ± 10.98	0.2132

DM: diabetes mellitus, BMI: body mass index, POD: postoperative day. * Chi-square test (Fisher’s exact *p*-value) for categorical variables and independent samples *t*-test for continuous variables.

**Table 4:** Logistic regression analysis.

	Univariate			Multivariate		
	aOR	95% CI	*p*-Value	aOR	95% CI	*p*-Value
DM	13.800		<0.0001	10.685	3.32–34.3	<0.0001
HTN	3.343		0.1486	0.415	0.056–3.053	0.3878
Phimosis grading						
Grade 0–1	Ref.		-	Ref.		-
Grade 2–3	1.920		0.4089	0.600	0.092–3.905	0.5933
Grade 4–5	2.711		0.0238	1.677	0.615–4.573	0.3127
Age	1.052		0.0327	1.007	0.953–1.065	0.7915
BMI	1.123		0.0002	1.061	0.984–1.143	0.1234

DM: diabetes mellitus, HTN: hypertension, BMI: body mass index, aOR = adjusted odds ratio.

## Data Availability

The datasets generated during and/or analyzed during the current study are available from the corresponding author on reasonable request.
